# The Variable Influence of Dispersant on Degradation of Oil Hydrocarbons in Subarctic Deep-Sea Sediments at Low Temperatures (0–5 °C)

**DOI:** 10.1038/s41598-017-02475-9

**Published:** 2017-05-22

**Authors:** Robert M. W. Ferguson, Evangelia Gontikaki, James A. Anderson, Ursula Witte

**Affiliations:** 1 Institute of Biological and Environmental Science, Oceanlab, University of Aberdeen, Newburgh, AB41 6AA UK; 2 Surface Chemistry and Catalysis Group, School of Engineering, University of Aberdeen, Aberdeen, AB24 3UE UK; 3 Department of Biological Sciences, University of Essex, Wivenhoe Park, Colchester, CO4 3SQ UK

## Abstract

The microbial degradation of petroleum hydrocarbons at low temperatures was investigated in subarctic deep-sea sediments in the Faroe Shetland Channel (FSC). The effect of the marine oil dispersant, Superdispersant 25 on hydrocarbon degradation was also examined. Sediments collected at 500 and 1000 m depth were spiked with a model oil containing 20 hydrocarbons and incubated at ambient temperature (5 and 0 °C, respectively) with and without marine dispersant. Treatment of sediments with hydrocarbons resulted in the enrichment of *Gammaproteobacteria*, and specifically the genera *Pseudoalteromonas*, *Pseudomonas*, *Halomonas*, and *Cobetia*. Hydrocarbon degradation was faster at 5 °C (500 m) with 65–89% of each component degraded after 50 days compared to 0–47% degradation at 0 °C (1000 m), where the aromatic hydrocarbons fluoranthene, anthracene, and Dibenzothiophene showed no degradation. Dispersant significantly increased the rate of degradation at 1000 m, but had no effect at 500 m. There was no statistically significant effect of Superdispersant 25 on the bacterial community structure at either station. These results show that the indigenous bacterial community in the FSC has the capacity to mitigate some of the effects of a potential oil spill, however, the effect of dispersant is ambiguous and further research is needed to understand the implications of its use.

## Introduction

The depletion of oil reserves onshore and in shallow waters has forced the industry to explore progressively deeper waters for as yet unexploited petroleum reserves. In addition, global warming has led to a dramatic shrinking of the sea ice in the Arctic, and hitherto inaccessible arctic oil reserves are now considered for exploration. Understanding the environmental implications of an oil spill in the cold and deep ocean is therefore urgent in order to improve oil contamination monitoring and optimise mitigation measures^1^. Most of our recent knowledge on the fate of hydrocarbons in the deep ocean stems from research following the catastrophic Deepwater Horizon (DWH) blowout in the Gulf of Mexico which occurred at a depth of 1500 m and resulted in the largest single marine oil spill to date. During DWH, approximately 4.9 million barrels of crude oil were released at depth and it has been estimated that between 2–15% of this oil was deposited onto deep-sea sediments by sedimentation of oil-contaminated marine snow^2, 3^. Once in contact with the sediment, hydrocarbons can become entrapped for long periods *via* absorption into sediment organics^4–7^ and impact sediment ecosystem function services^8–13^. The persistence in sediments of hydrocarbons recalcitrant to degradation may also have negative impacts on sediment communities biodiversity and pose significant health risks as accumulated hydrocarbons slowly enter the food web^14^.

Microbial biodegradation is a key process for the removal of oil from the marine environment^15–18^. Hydrocarbon-degrading microbes exist as part of the rare biosphere in the ocean, forming “seed populations” that can respond rapidly to hydrocarbon exposure^19^. In the case of DWH, a succession of hydrocarbon-degrading bacteria (HDB) including *Colwellia, Cycloclasticus*, *Marinobacter* and *Pseudomonas*, contributed an important ecosystem service via biodegradation of hydrocarbons in the sediments and water column^20–26^. Oil-polluted deep sea sediments, in particular, also became enriched in sulfate-reducing, anaerobic bacterial families *Desulfobacteraceae* and *Desulfobulbaceae* as a result of oil-derived marine snow sedimentation and the occurrence of anaerobic microniches^24^. In an attempt to mitigate the effects of the DWH oil spill to coastal areas and enhance biodegradation rates, the dispersant COREXIT (9500 A and EC9527A) was applied to surface waters as well as directly into the wellhead at 1500 m below surface level. The effect and persistence of COREXIT in the environment, including its impact on the indigenous microbial communities in the Gulf remain unclear^9, 27–29^. The application of dispersants may result in enhanced transport of oil hydrocarbons deeper into the sediment, where anoxic conditions reduce degradation rates^30^. On the other hand, dispersants may result in faster bacterial degradation by increasing the surface area of the oil droplets^31^. There is, however, no consensus in the literature with some studies reporting faster, slower or no change in degradation rates in the presence of a dispersant^9, 27, 28, 32^. Deep water oil and gas exploration in the Faroe-Shetland Channel (FSC) currently occurs down to 1100 m depth (Rosebank oil field). In keeping with many deep-water and arctic oil reservoirs, the FSC remains underdeveloped due to technical challenges associated with oil extraction, including its remoteness, depth, geology and rough weather^33, 34^ but maturing North Sea oilfields and improving technology for extracting oil in deep water will likely lead to increased exploitation in the future. The FSC is characterised by a complex hydrography with northward-flowing warm North Atlantic waters overlying cold water masses of Arctic origin flowing southwards. As a result, water temperature varies dramatically within only a few hundred meters, from ~8 °C in the top 200 m to sub-zero temperatures below 600 m^35^. In addition, strong near-bottom current velocities and low sediment deposition have created generally coarse-grained seafloor sediments in the FSC in contrast to typically muddy sediments prevailing in most deep-sea environments at a similar depth. The subzero temperatures, complex hydrography and unusual sedimentary environment, as well as rough weather conditions and remoteness of the FSC, suggest that in the event of a large oil spill, mitigation measures typically used in the event of a large oil spill may prove inadequate, and compromised further by delays caused by rough weather conditions and remoteness of the FSC^36^. It is thus necessary to obtain system-specific data on the ecosystem response to current remediation techniques as well as an assessment of its natural capacity for biologically-mediated hydrocarbon degradation in order to appraise the ecological impact of a potential oil spill and assess suitable oil spill response strategies.

The aim of this study was to quantify hydrocarbon degradation in the deep-sea sediments of the FSC, to characterise the indigenous HDB community, and investigate the effect of a marine oil dispersant on degradation rates. This was done by incubating sediments collected from the FSC at 500 and 1000 m (subsequently referred to as stations FSC500 and FSC1000 respectively, Supplementary information 1) with a model oil comprising a mixture of 20 hydrocarbons (Supplementary Fig. 1) for 50 days, with and without marine dispersant in aerobic conditions (Supersidpersant 25, Oil Technics Ltd., Aberdeen, UK). A hydrocarbon mixture was used as it is more representative than using single hydrocarbons, which may miss synergistic effects of components, but in contrast to a crude oil, gives a consistent mixture that can be compared between experiments. Degradation of the model oil components was quantified by GC-FID and the microbial community was characterised with shotgun sequencing of the 16S rRNA gene. To our knowledge, this is the first study to provide a baseline description of the naturally occurring HDB community at a sub-arctic deep-sea site exposed to subzero temperatures and its intrinsic capability to degrade hydrocarbons. Lack of such baseline data has been repeatedly highlighted as an obstacle in assessing the environmental effects of oil spills in post-DWH studies^19, 37, 38^. Our results are of relevance to oil degradation in the Arctic due to the particularly low bottom water temperatures and the arctic origin of the water masses in our study area.

## Results

### Degradation of aliphatic hydrocarbons

**Figure 1:**
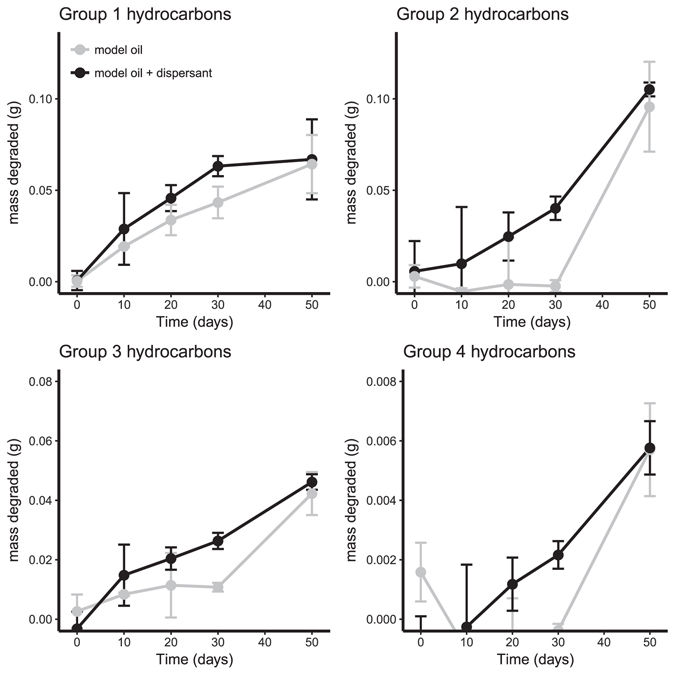
Mass of each aliphatic hydrocarbon group degraded during slurry incubations in FSC500 at 5 °C. Error bars are standard deviation, n = 3. Black lines are model oil and grey are model oil + dis.

**Figure 2:**
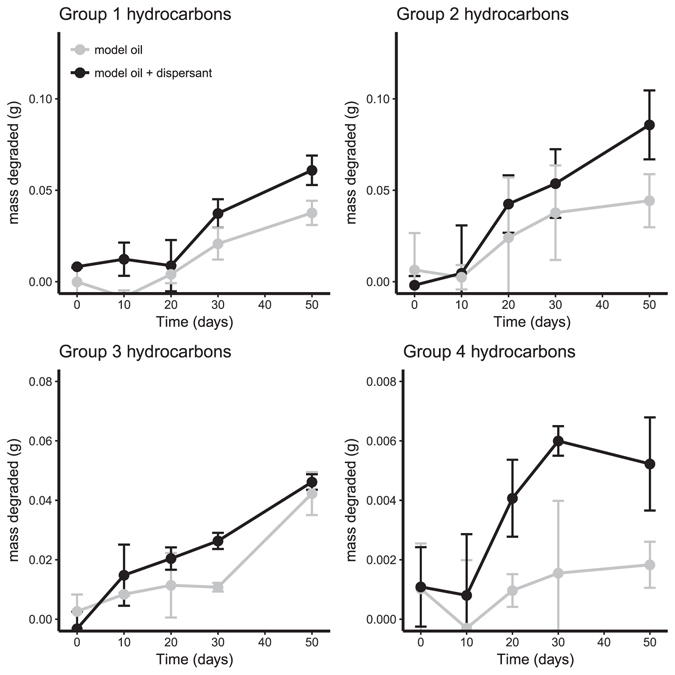
Mass of each aliphatic hydrocarbon group degraded during slurry incubations in FSC1000 at 0 °C. Error bars are standard deviation, n = 3. Black lines are model oil and grey are model oil + dis.

At 5 °C (FSC500), the concentration of all aliphatic hydrocarbon groups declined by 65–82% after 50 days of incubation (Fig. 1, Table 1). The degradation of aliphatics proceeded slower at 0 °C (FSC1000) with just 26–38% decrease in concentration by the end of the incubation period (Fig. 2, Table 1). The effect of dispersant was not consistent between stations. Dispersant had no effect on the final proportion of aliphatic hydrocarbons degraded after 50 days at 5 °C, however, it did eliminate the lag time before the onset of degradation in Groups 2, 3, and 4 (Table 1 and Fig. 1). In contrast to 5 °C the total amount of aliphatic hydrocarbons degraded after 50 days at 0 °C was significantly higher with dispersant for all groups (Fig. 2, Table 1) and ranged between 62–75% (compared to 26–38% without dispersant).

**Table 1:** Summary of total hydrocarbon degradation for each group after 50 days and results of comparison between rates between model oil and model oil + dis treatments with ANCOVA. n = 15 with 3 per time point for ANCOVA and n = 3 for total degradation after 50 days.

Station	Group	% degraded after 50 days			Rate comparisons with ANCOVA	
		Model Oil	Model Oil + Dis	significance	Model Oil v Model Oil + Dis	
					R^2^	Significance
Aliphatic						
FSC500	1	65.6 ± 16.3	68.3 ± 22.3	0.859	0.76	0.0585
	2	81.0 ± 20.8	89.0 ± 3.2	0.543	0.69	0.0614
	3	78.6 ± 13.5	85.7 ± 4.9	0.413	0.80	0.0189
	4	82.3 ± 22.5	83.2 ± 13.0	0.966	0.69	0.0319
FSC1000	1	38.4 ± 6.8	62.2 ± 8.2	0.0449	0.69	0.0319
	2	37.5 ± 12.3	72.6 ± 16.0	0.0429	0.61	0.0310
	3	34.6 ± 11.6	66.5 ± 1.8	0.0134	0.56	0.1160
	4	26.4 ± 11.2	75.4 ± 22.6	0.0486	0.53	0.0372
Aromatic						
FSC500	Naphthalene	79.7 ± 7.4	85.1 ± 6.8	0.417	0.83	0.241
	Fluorene	83.3 ± 17.1	75.3 ± 25.2	0.670	0.62	0.248
	Phenanthrene	88.8 ± 13.0	90.2 ± 7.6	0.885	0.64	0.199
	Anthracene	68.8 ± 27.3	79.6 ± 35.3	0.119	0.56	0.179
	Fluoranthene	82.3 ± 18.6	89.8 ± 5.0	0.521	0.62	0.166
	Dibenzothiophene	80.3 ± 21.7	74.4 ± 24.7	0.777	0.34	0.124
FSC1000	Naphthalene	20.4 ± 3.5	69.4 ± 3.4	0.0006	0.51	0.0226
	Fluorene	28.7 ± 10.4	78.8 ± 13.3	0.0212	Not comparable	
	Phenanthrene	46.9 ± 1.3	85.3 ± 9.6	0.0115	0.59	0.141
	Anthracene	15.7 ± 4.0	70.6 ± 28.5	0.0815	Not comparable	0.211
	Fluoranthene	10.2 ± 5.4	85.6 ± 19.7	0.0151	Not comparable	0.0249
	Dibenzothiophene	−1.9 ± 3.3	74.0 ± 17.4	0.0105	Not comparable

### Degradation of PAHs

**Figure 3:**
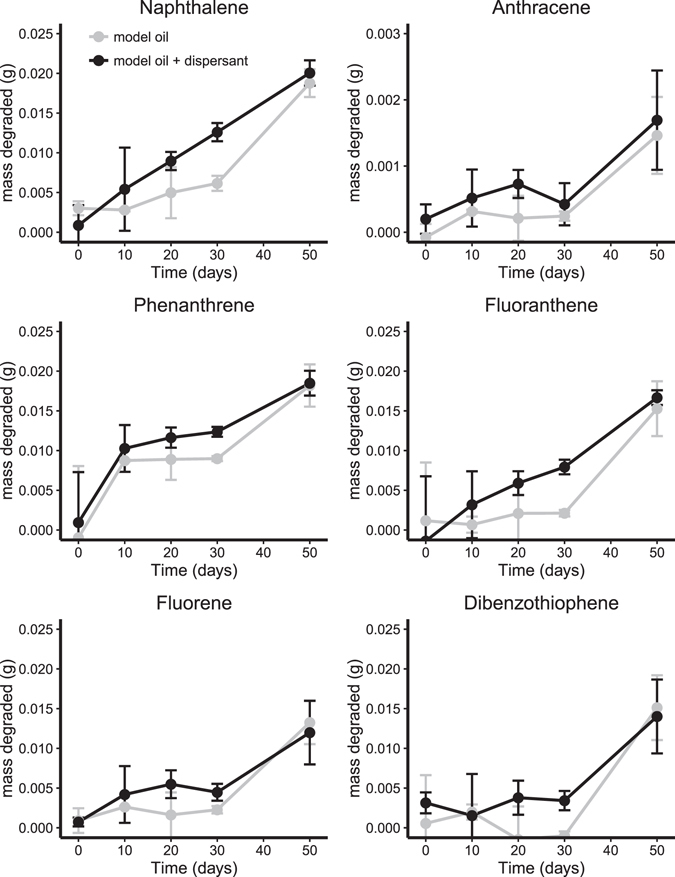
Mass of each PAH degraded during slurry incubations in FSC500 at 5 °C. Error bars are standard deviation, n = 3. Black lines are model oil and grey are model oil + dis.

**Figure 4:**
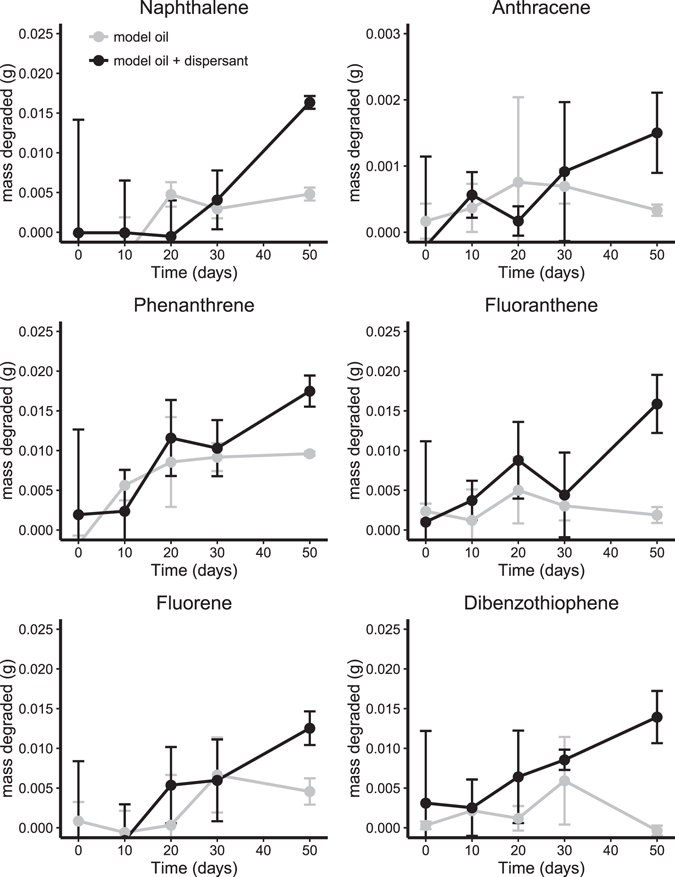
Mass of each PAH degraded during slurry incubations in FSC1000 at 0 °C. Error bars are standard deviation, n = 3. Black lines are model oil and grey are model oil + dis.

The degradation of total PAHs after 50 days at 5 °C (FSC500) was considerable (up to 90% removal) independent of the presence of dispersant (Table 1, Fig. 3). PAH degradation at 0 °C after 50 days ranged between 69–85% with dispersant while little (naphthalene, fluorene and phenanthrene) or no (anthracene, fluoranthene, and dibenzothiophene) degradation was observed in the absence of dispersant (Table 1, Fig. 4).

### Background FSC sediment community

The natural background bacterial communities from both stations were dominated by Proteobacteria (81% of all reads) of which the class *Gammaproteobacteria* was the most dominant (63% of all reads). Only 15% of the OTUs were shared between the two stations and permutational multivariate analysis of variance (PerMANOVA) revealed that the stations were significantly different to each other (p = 0.008, R^2^ = 0.26, n = 5) (Supplementary Fig. 2).

### Microbial dynamics during hydrocarbon degradation

**Figure 5:**
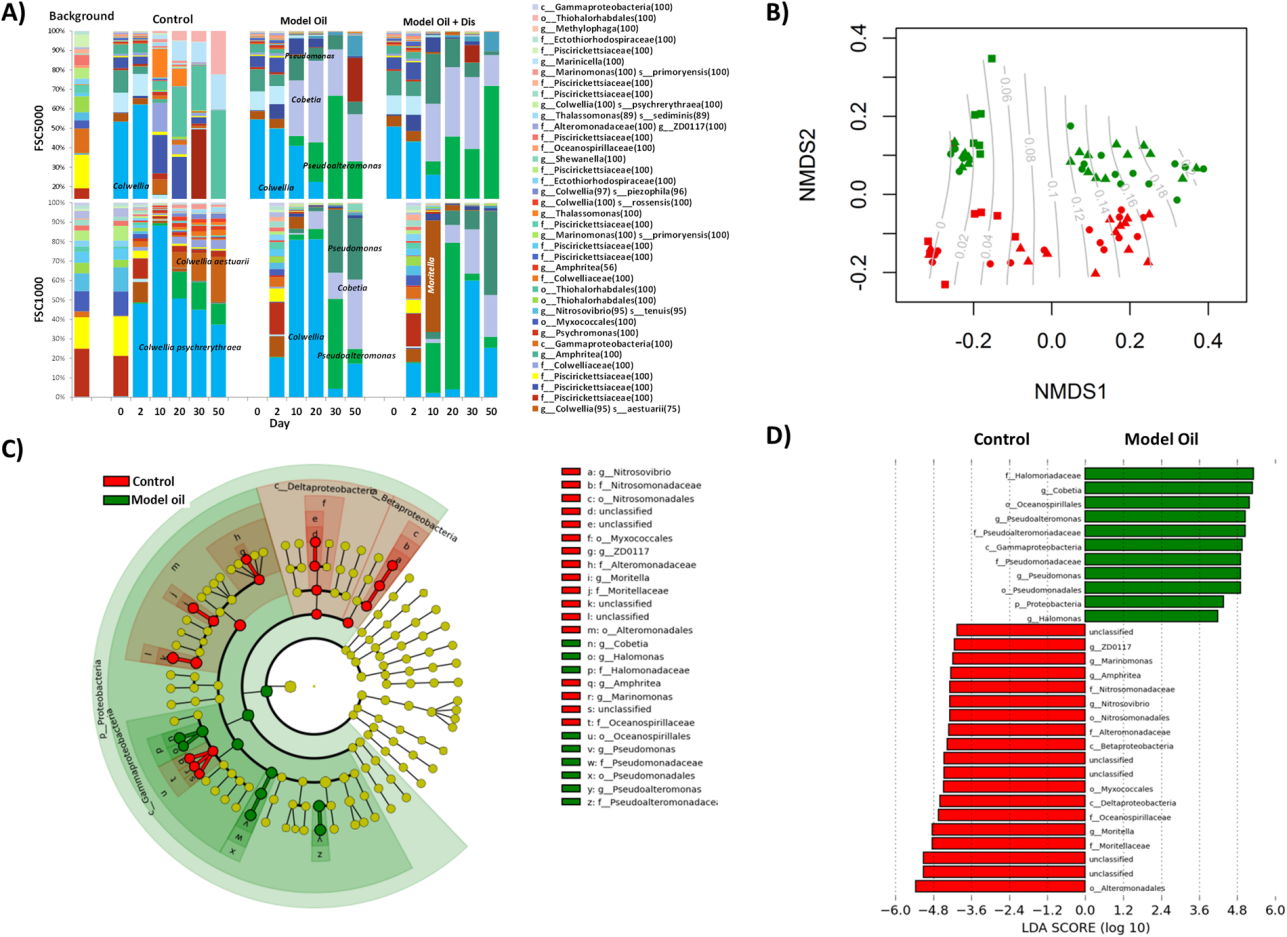
Summary of bacterial communities in slurry incubations: Panel A) Relative abundance of top 50 OTUs (No sample collected for T0 FSC1000 model oil treatments as they did not differ from control T0). Panel B) nMDS based on Jaccard index; red = FSC1000 and green = FSC500; square points = control, triangles = oil + dis, and circles = oil only; contours show total mass of hydrocarbon degraded, predicted by ordisurf(). Panel C) Cladogram output from LefSe analysis showing consistently differing taxonomic groups between oil treated and control slurry incubations. Panel D) LDA effect scores for output from LefSe analysis. For panels C and D red = control and green = model oil.

There was a shift in bacterial OTUs in all treatments during the incubation experiment (Fig. 5a), evidenced by the significant explanatory power of time in perMANOVA analysis (p = 0.001, R^2^ = 0.04, N = 77). The presence of model oil modified the shift of bacterial OTUs (p = 0.001, R^2^ = 0.2, perMANOVA, N = 77) and the mass of hydrocarbon degraded was significantly predicted by ordisurf()(See Methods, Statistical Analysis) for position on the nMDS (p = 0.001, R^2^ = 0.4, N = 77) with a trajectory from left to right corresponding to higher mass of hydrocarbon degraded (Fig. 5b). Superdispersant 25 did not have a significant effect on the bacterial community (p = 0.08, R^2^ = 0.01, perMANOVA, N = 77) but sampling location did (p = 0.002, R^2^ = 0.06, perMANOVA, N = 77) reflecting the differing natural communities used as starting points (Supplementary Fig. 2). The station effect persists through the experiment, despite the similarity in the dominant OTUs in the model oil treated incubations and is visualised as a separation between the stations in the y-axis of the nMSDS plot (Fig. 5b).

In FSC1000 the natural background community was still present at the start of the incubations (day 0 and 2) however, in FSC500 there was a notable shift in the bacterial community structure between the natural background and at the start of the incubations (Fig. 5a). Subsequently to this, the bacterial OTUs shifted in a similar fashion for both stations, indicating that the natural oil degraders were not lost from FSC500 during transport and storage.

The control incubations in both stations were initially dominated by *Colwellia psychrerythraea*. This OTU remained dominant in FSC1000 until the end of the study whereas in FSC500 it was largely replaced by OTUs from the orders Marinicellales and Thiohalorhabdales. OTUs related to *Colwellia aestuarii* and *Pseudoalteromonas* were also predominant in FSC1000 controls from day 30 onwards but did not replace *Colwellia psychrerythraea*. In the model oil-treated incubations, *Colwellia psychrerythraea* was also initially dominant. However, bacterial communities in oil treatments diverged from those in controls with time and OTUs from the genera *Pseudoalteromonas, Cobetia*, and *Halomonas* predominated as hydrocabons were degraded. This shift was delayed in the FSC1000 model oil only treatment reflecting the later start in oil degradation (Figs 1–4). There were no specific OTUs related significantly to the presence of Superdispersant however, by causing the degradation process to proceed faster, the presence of dispersant affected the timing of bacterial succession.

### Links between bacterial taxa and hydrocarbon degradation

LEfSe analysis (See Methods, Statistical Analysis) was carried out to identify which taxa were significant biomarkers of hydrocabon degradation (Fig. 5c,d)^39^. This revealed general shifts at the class level with *Gammaproteobacteria a*ssociated with the model oil treatments and *Beta/Deltaproteobacteria* associated with the control. At a lower taxon level, the *Beta/Deltaproteobacteria* in controls was *Nitrosomonas* and *Myxococcales*. LEfSe also revealed differences between the control and model oil groups at lower taxonomic groups within *Gammaproteobacteria*. The order *Alteromonas* (specifically the genera *ZD011*, *Moritella*, and *Colwellia*) was dominant in controls while *Oceanospirillales* was associated with the hydrocabon treatments. Within the *Oceanospirillales* order, the genera *Cobetia* and *Halomonas* were associated with model oil while *Marinomonas* and *Amphritea* were associated with the control. The genera *Pseudomonas* and *Pseudoalteromonas* were also associated with model oil.

## Discussion

Despite the wealth of information on the biotic response and environmental consequences of oil release and dispersant use in the deep sea following DWH, our knowledge on the environmental factors that regulate microbial hydrocarbon degradation and bioremediation efficiency remains incomplete^19^. Here, the first data is presented on the structure and function of HDB communities in the deep FSC, an area of active exploration and exploitation of oil reservoirs in deep waters and of particular oceanographic importance due to its role in the global thermohaline circulation. Furthermore, we investigated the effect on hydrocarbon degradation of the marine dispersant, Superdispersant 25, which is approved by the UK Food and Environment Research Agency and the most likely to be employed in the event of an oil spill in UK waters. Results showed that, without dispersant, the degradation of all hydrocarbons was considerably higher at 500 m (5 °C) compared to that at 1000 m (0 °C). Lower biodegradation of alkanes and PAHs at 0 °C compared to 5 °C has also been observed in seawater incubations from a Norwegian fjord^40^. It is not possible from results here to determine whether higher biodegradation at 500 m is purely due to the higher temperature or if differences in the bacterial community and sediment characteristics also contributed. However, basin-wide investigations have recently identified temperature as the main factor determining the oil biodegradation potential of bacterial communities in geographically separated oil-polluted sediments in the Mediterranean^41^. Regardless, our results demonstrate that oil will persist longer in the deeper sediments of the FSC and suggest that the impact of oil release in the deep FSC or contamination of deep-water sediments as a result of water mass circulation would be higher due to the sub-zero temperatures prevailing below 600 m.

Biodegradation of the hydrocarbon mixture after 50 days in 0 °C incubations was lower than that measured in arctic seawater incubations with a medium grade crude oil at −1 °C over 60 days^42^; in the latter case 58–61% of total measurable oil was lost over the incubation period and specific components (heptadecane, octadecane, naphthalene, phenanthrene, dibenzothiophene) reached complete degradation^42^. In our study, the same components showed less than 50% degradation after 50 days (Table 1) with the most extreme difference observed in dibenzothiophene degradation which was zero. It is, therefore, reasonable to suggest that PAHs, such as fluoranthene, anthracene, and Dibenzothiophene, may become entrapped in the subsurface sediments of the deep FSC and could be periodically released by bioturbation or incorporated into faunal biomass resulting in long term impacts. Hydrocarbon degradation without Superdispersant 25 at 5 °C after 50 days was >75% for both aliphatic and aromatic hydrocarbons (except anthracene, Table 1) with most activity observed after 30 days. This lag period in the onset of degradation may be the reason why Baelum *et al*.^9^ found merely 25% removal of total hydrocarbons in 5 °C incubations of Gulf of Mexico deep seawater with Macondo oil after 20 days. Higher biodegradation of alkanes and PAHs (~90%) at 5 °C were measured by Campo *et al*.^32^ in 42-day incubations with light crude but these experiments were performed in artificial seawater inoculated with an enriched oil-degrading bacterial culture which could have accelerated the degradation process^43^. The environmental lifetime of hydrocarbons in FSC sediments predicted by linear regression ranged between 2 and 5 months (at 5 °C and 0 °C respectively, Supplementary Table 4), at least for those components subject to bacterial degradation. It should be noted however that degradation rates from laboratory incubations (although a vital tool) cannot be extrapolated to natural ecosystems without caution^8^. Hydrocarbon degradation appears to progress much slower *in situ*; in comparison, Liu *et al*.^44^ found only light to moderate ( < 25%) weathering of oil in heavily contaminated sediments 1 year after the DWH spill and subsequent studies in the Gulf of Mexico have confirmed the presence of Macondo oil in deep-water sediments 4 years after the DWH spill although reduced in mass by 80–90% compared to levels immediately after contamination^45^. Migration of oil in deeper sediment layers where anaerobic conditions prevail may partly explain slower biodegradation rates in the field^30, 46^. In the case of the Exxon Valdez and the West Falmouth oil spills, hydrocarbons have been shown to persist for decades after contamination in the subsurface layers of coastal sediments^47, 48^.

Hydrocabon contamination of FSC sediments here resulted in the enrichment of *Gammaproteobacteria*, specifically the order *Oceanospirillales and the genera Pseudoalteromonas* and *Pseudomonas*. *Oceanospirillales* and *Pseudomonas* taxa also became enriched in the DWH deep water plume and were most abundant during the initial, unmitigated flow phase of the spill when the concentration of hydrocarbons and the fraction of insoluble n-alkanes and cycloalkanes were highest^20, 49, 50^. The enriched *Oceanospirillales* taxon in the DWH plume was an unclassified and uncultivated OTU within the family *Oceanospirillaceae* ^20^ whereas in our study the enriched taxa belonged to *Halomonadaceae*. Therefore, although at higher taxonomic levels, such as class and order, the main HDB may be similar between geographic locations; site-specific research is required for fine low taxon detail.

The LEfSe analysis identified two biomarker OTUs within the *Halomonadaceae*, *Cobetia* and *Halomonas*, which were associated with hydrocabon contamination in our incubations. The genus *Cobetia* has been previously isolated from oil-contaminated coastal waters of the Persian Gulf and is known to produce biosurfactants and degrade hydrocarbons including phenanthrene and dibenzothiophene which were present in the model oil mixture^51, 52^. However, *Cobetia* was not identified among HDB in the Gulf of Mexico. *Halomonas* was one of the dominant HDB in contaminated surface waters and, to a lesser extent, the deep water plume during the DWH spill as revealed by DNA stable isotope probing and cultivation-based methods^53^. The relative abundance of *Pseudoalteromonas*, which also became enriched in our model oil incubations, increased in the DWH deep water plume during partial capture as petroleum hydrocarbon concentrations decreased and the more dilute plume consisted of more BTEX relative to alkanes^20^. The decrease of ZD0117 in oil-enriched treatments in this study has also been observed in Antarctic seawater samples following contamination with oil^54^. The genus *Colwellia* is thought to play a predominant role hydrocarbon degradation in the deep sea. This is supported by the psychrophilic lifestyle^55^ and the ability of members of *Colwelliaceae* to produce EPS under low temperature and high pressure^56^ along with the metabolic potential for hydrocarbon degradation^50^. Indeed, *Colwellia* was one of the major taxa that became enriched in the DWH deep water plume and was the dominant bacteria in flocs and contaminated deep water sediments in the Gulf ^9, 10, 57^. In this study, *Colwellia* was one of the most abundant taxa in both control and hydrocabon-contaminated incubations. This is not surprising since *Colwellia* is a heterotrophic group that does not depend solely on external hydrocarbon supply for growth. Yang *et al*.^24^ reported the presence of *Colwellia* in both oily and non-oily Gulf sediments collected 5–7 months after the DWH spill (September, October and November 2010) and also concluded that *Colwellia* could be autochthonous to deep surficial sediments and cannot be unambiguously linked to oil contamination. The fact that *Colwellia* was abundant in control incubations here but not in the natural background samples may suggest that it is a strong competitor in enrichments/microcosms. There was a shift in the FSC500 bacterial community between background and day 0 incubations, while this was not the case in FSC1000 (Fig. 5). The shift is mainly related to the genre *Colwellia*, further demonstrating its robustness during sampling and storage, and could be attributed to the longer interval (one week) between sampling FSC500 and initiation of the incubation experiment compared to FSC1000. Nevertheless, the similar response of bacterial communities in FSC1000 and FSC500 to hydrocarbon contamination suggests that results are representative. It is however important to stress that though there are many benefits to the microcosm strategy employed in this study, not least statistical power gained from replication and destructive sampling, many questions remain as to how hydrocarbon degradation would proceed in the field. This study should be seen as a first step and further study in the field is required.

Studies on dispersants have generated confounding results, most likely because of the non-specific metrics used to define biodegradation (e.g. mineralisation to CO_2_, chemical disappearance quantified by GC-MS) and differences in the state and concentration of oil, as well as concentration and perhaps most significantly type of dispersant used in laboratory incubations^28, 29^. Previous studies about microbial dispersant impacts have generally focused on Corexit, the dispersant used after DWH. In this study we used Superdispersant 25, as it is approved for used in the FSC by the EU and UK and very little is known about its environmental effects. We do not know to what extent the effects of Corexit can be compared with Superdispersant 25, and to our knowledge this is the first study to investigate its effects on the degradation of hydrocarbons. It is known that Superdispersant 25 is toxic to a range of marine animals, both with and without the presence of hydrocarbons^58, 59^, although its toxicity is less than that of Corexit 9527^59^, In the case of Corexit 9500, hydrocarbon degradation by deep water bacterial communities in the Gulf of Mexico (~5 °C) was either unaffected^32^, enhanced^60^ or even inhibited^27^. In the latter case, suppressed degradation of certain classes of hydrocarbons, particularly alkanes, was associated with lower bacterial protein synthesis and exoenzyme activities in dispersant treatments. Arctic seawater microorganisms from the Chukchi Sea degraded similar amounts of oil at −1 °C with and without the dispersant Corexit 9500^42^. In this study with Superdispersant 25, we observed both no difference (5 °C) and significantly increased (0 °C) amount of hydrocarbons degraded by day 50. The presence of dispersant diminished the effect of temperature on the relative rates of hydrocarbon degradation, i.e. the amount of aliphatic and aromatic hydrocarbons degraded after 50 days was comparable between 0 °C and 5 °C when dispersant was present whereas degradation was significantly depressed at 0 °C without dispersant. Despite the apparent lack of impact of dispersant on the final amount of hydrocarbons degraded at 5 °C, the onset of degradation of several less soluble compounds was initiated earlier when dispersant was present (hydrocarbon groups 2, 3, and 4). In contrast, more soluble hydrocarbons (n-decane and 1-decene, Group 1) exhibited similar degradation patterns with and without dispersant suggesting that only the less water soluble hydrocarbons have their bioavailability increased by Superdispersant 25. Nevertheless, it seems that the effect of dispersant on degradation is specific for individual hydrocarbons^25, 61^ and depends on the characteristics of the dispersant to be used^61^. These observations highlight the challenge of predicting the consequences of dispersant use to mitigate oil spills in the natural environment where a number of physicochemical and biological factors may also contribute to hydrocarbon biodegradation^43, 62^. Differences in hydrocarbon degradation patterns between treatments here were not accompanied by shifts in the bacterial community structure that could be specifically linked to dispersant application, although there could have been differences at species, strain or genome level not detected with our sequencing strategy. Previous studies have demonstrated taxa-specific and concentration-dependent effects of dispersants on microorganisms; certain *Colwellia* and *Alcanivorax* taxa, for example, responded to dispersants or oil-dispersant mixtures^9, 27, 63, 64^ whereas *Marinobacter* ^27^ and *Acinetobacter* ^63^ were suppressed by dispersants. In contrast to most studies, however, relatively low concentrations of hydrocarbons and Superdispersant 25 were applied in our incubations to simulate marine biodegradation of chemically dispersed oil at the low end of the range of dispersant concentrations found after a spill, which could explain the lack of effect of Superdispersant 25 on the bacterial community composition^65^.

Overall, this study is the first to demonstrate the capability of deep water, subarctic sediment microbial communities to degrade hydrocabons at temperatures of 0 and 5 °C. Hydrocarbon degradation was dependent on temperature with significantly slower degradation of both aliphatic and aromatic hydrocarbons at 0 °C compared to 5 °C. Certain PAHs, such as fluoranthene, anthracene and dibenzothiophene did not show detectable levels of degradation at 0 °C suggesting long-term impacts of oil contamination at near zero or subzero temperatures. The bacterial community response was consistent between 500 and 1000 m with the class *Gammaproteobacteria*, orders *Oceanospirillales* and *Pseudomonadales*, and genera *Pseudoalteromonas*, *Pseudomonas*, *Halomonas* and *Cobetia* predominating in model oil-contaminated treatments. The high degree of consistency between stations in terms of bacterial response to the model oil provides encouragement that oil contamination could be monitored by proxy of bacterial changes in the FSC. Robust and highly portable routine molecular methods, such as qPCR, could be developed to detect such changes. Due to the Arctic origin of bottom water masses in our study area, results here are relevant to oil biodegradation efficiency and bioremediation potential of bacterial communities in Arctic ecosystems where the ongoing exploration for oil and gas in offshore areas and a growing interest in developing the Northern Sea Route (NSR) as an alternative transportation route increase the risk of hydrocarbon pollution.

## Methods

### Sediment collection

Sediment was collected from two stations at 538 and 994 m (subsequently referred to as stations FSC500 and FSC1000, respectively) in the FSC using a day grab (Supplementary information 1). Sampling was conducted on the *FRV* Scotia (cruise number Sc201405) between 24/04/14–08/05/14. Sediment from five grabs collected at each station was pooled and stored at 1 °C under approximately 10 cm of ambient seawater. The overlying water was constantly aerated with an aquarium air pump (Airvolution AV2, Interpet, UK) and air stones until slurry incubations were initiated. Additionally, approximately 10 g of sediment from each grab were immediately stored at −80 °C for molecular analysis. Experiments were initiated after returning from the cruse, so were stored for 14–21 days before the start of the experiment, control incubations were run so we could account for any changes in the community due to storage and transport.

### Preparation of Model oil

A model oil containing 20 hydrocarbons was formulated with a mixture of aliphatic, aromatic, polyaromatic (PAH), and resin components proportions are shown in Supplementary Fig. 1. The density of the model oil was 880 kg/m^3^, equivalent to a medium crude oil. The model oil was based on hydrocarbon molecules containing more than 8 carbon atoms as it is thought that these components are more likely to be deposited on the seabed^2, 66^. Although olefins/alkenes are present in crude oil (see ref. 67) these are usually present at lower concentrations than employed in the model oil prepared here. Our model was loosely based on the composition of a North sea crude (Schiehallion) however, this had to be manipulated in such a manner as to ensure mutual solubility of all components.

### Experimental procedure

Sediment from each station was incubated with either model oil alone or model oil and marine dispersant, Superdispersant 25 (Oil Technics Ltd., Aberdeen, UK) (Supplementary Table 1). The treatments are hereafter referred to as model oil (model oil only) and model oil + dis (model oil and dispersant). Each treatment was run in triplicate for each time point with a live control (sediment and seawater only) and sterile control (autoclaved sediment with model oil or model oil + dis) run in parallel. To prepare the incubations, 15 ml of ambient seawater was added to the vials which were then sterilised by autoclaving at 121 °C and 100 kPa for 15 min. After autoclaving, 5 ml of sediment was added to each vial and, depending on treatment, was spiked with 0.44 g of model oil and 0.015 g Superdispersant 25 (ratio of 30:1 oil: dispersant to mimic application at blowout location in accordance with manufacturer’s instructions). The vials were then incubated in the dark on a shaker table at 150 rpm at 5 °C or 0 °C for FSC500 and FSC1000, respectively (reflecting the *in situ* temperature at each station). On days 10 and 30, the headspace in each vial was replenished with sterile air through the septa to ensure the conditions remained aerobic. Destructive sampling was carried out on days 0, 2, 10, 20, 30, and 50 for all treatments. Approximately 2 ml of the sediment were collected aseptically and stored at −80 °C until molecular analysis. The remainder was stored in the glass incubation vial at −20 °C until GC analysis.

### Hydrocarbon extraction and analysis

To determine the extent of hydrocarbon loss due to evaporation, 1 g of model oil was incubated in glass vials (n = 3) at room temperature. A decrease in mass of less than 1% was detected after 50 days. Hydrocarbons were extracted from the slurries by liquid-liquid extraction with 3 × 10 ml dichloromethane (DCM) (VWR, UK). 1 µl of the DCM fraction was then subjected to GC analysis using toluene as an internal standard to correct for injector error (20 µl/ml toluene). Samples were analysed on a Varian CP3800 fitted with 30 m Zebron ZB-50 column and FID detector. The hydrocarbons were quantified against an external standard containing known amounts of the model oil components. The conditions for the analysis were as follows; injector and detector temperature was 330 °C and the injection split ratio was 80:1, initial oven temperature was 50 °C with a 3 min hold and then increased at 10 °C min^−1^ to 110 °C, followed by an increase to 200 °C at 5 °C min^−1^ with at 12 min hold, and finally ramping to 300 °C at 20 °C min^−1^. All components showed recovery rates 90–95% after 50 days in the sterile controls (Supplementary Table 2). The exception to this was ethylbenzene and xylenes which showed recovery levels of < 75%. The partial loss of these specific components cannot be accounted for at this stage but probably reflect the greater relative volatility of these components and have been excluded from further analysis.

### Microbial community analysis

Total DNA was extracted using the FastDNA™ SPIN Kit for Soil (MP biomedicals, Cambridge, UK) from 0.4 g of sediment according to the manufacturer’s instructions. Pair ended (300 × 2) amplicon sequencing across the V3–V4 variable region of the 16 S rRNA gene was carried out on the Illumina MiSeq platform using the following primers: Forward Primer = 5′ TCGTCGGCAGCGTCAGATGTGTATAAGAGACAGCCTACGGGNGGCWGCAG, Reverse Primer = 5′ GTCTCGTGGGCTCGGAGATGTGTATAAGAGACAGGACTACHVGGGTATCTAATCC. Approximately 13 M reads were obtained with a read depth 30–110 K per sample (88 samples total). Bioinformatics analysis was carried out on the Maxwell high performance computing cluster at the University of Aberdeen, using Mothur v 1.36.1^68, 69^ and associated resources (PandaSeq^70^, Precluster^71^, SILVA^72^, Greengenes^73, 74^, RDP Naive Bayesian classifier^75^, and UCHIME^76^) as outlined in Drumbell *et al*.^77^ see Supplementary Information 2. Sequences from this study are available through the European Nucleotide Archive under project PRJEB15950 (ERP017791).

### Statistical analysis

Statistical analysis was carried out in R^78^ and the package Vegan^79^.

To simplify results, hydrocarbons that had similar degradation profiles were grouped by principal component analysis. The raw data for individual components is given in Supplementary Data 1. Group 1 comprised the two shortest chain aliphatics, the alkane n-decane and the alkene 1-decene. Group 2 comprised the medium length aliphatic hydrocarbons tetradecane, pentadecane, hexadecane, heptadecane, icosane, and docosane. Group 3 comprised the aliphatic alkane dodecane, and the alkene octadecene. Group 4 contained tetracosane (Supplementary Table 3). To test whether degradation rates were significantly different between conditions, analysis of covariance (ANCOVA) was used to compare the slopes of the linear regressions of hydrocarbons degraded over time with the formula “hydrocarbon mass ~ day * treatment”. Significantly different degradation rates were accepted at p < 0.05, or if slopes were incomparable (i.e. did not follow the same x, y relationship) comparison for sterile versus live incubations is given in Supplementary Table 4.

To correct for variation in sequencing depth between samples, rarefaction was carried out using the r function “rrarefy()”. For beta diversity a distance matrix was created with the function “vegdist()” using the Jaccard index. This was visualised with non-metric multidimensional (nMDS) scaling using the function “metaMDS()”. To test for differences between sampling groups, permutational multivariate analysis of variance (perMANOVA) using distance matrices was carried out using the function “adonis()” with 1000 restarts. To check if hydrocarbon concentrations were correlated with the nMDS, ordisurf()was used to fit vectors to the surface ordination plot with generalized additive models. Significance was accepted at p < 0.05. Linear Discriminant Effect Size analysis (LEfSe) was used to identify bacterial shifts associated with different treatment conditions using the Galaxy pipeline (available at https://huttenhower.sph.harvard.edu/galaxy/)^39^. First LEfSe identifies features (e.g. clades or OTUs) that are significantly differently abundant between groups with non-parametric factorial Kruskal-Wallis sum-rank tests; it then interrogates these features for biological consistency and relative importance with Wilcoxon rank-sum tests among subclasses and Linear Discriminant Analysis to determine the effect sizes.

### Data Availability

Sequences from this study are available through the European Nucleotide Archive under project PRJEB15950 (ERP017791). All other data generated or analysed during this study are included in the Supplementary Information file and tables.

## Electronic supplementary material

Supplementary Information

Dataset 1
