# Supplementary material for: The Variable Influence of Dispersant on Degradation of Oil Hydrocarbons in Subarctic Deep-Sea Sediments at Low Temperatures (0–5 °C)
Source: Sci Rep. 2017 May 22;7:2253. doi: 10.1038/s41598-017-02475-9 (PMC5440406; doi:10.1038/s41598-017-02475-9)
Supplement: Supplementary file 1 — Supplementary Information [file 41598_2017_2475_MOESM1_ESM.pdf]

Supplementary Information

The Variable Influence of Dispersant on Degradation of Oil Hydrocarbons in Subarctic Deep-Sea Sediments at Low Temperatures (0-5°C).

Robert Ferguson [1,3*], Evangelia Gontikaki[1], James A. Anderson[2], Ursula Witte[1]

[1]Institute of Biological and Environmental Science, Oceanlab, University of Aberdeen, Newburgh, AB41 6AA, UK

[2]Surface Chemistry and Catalysis Group, School of Engineering, University of Aberdeen, Aberdeen AB24 3UE, UK

[3]Current address: Department of Biological Sciences, University of Essex, Wivenhoe Park, Colchester, CO4 3SQ, UK

*Corresponding author: Robert Ferguson (rmwfer@essex.ac.uk)

Contents

Supplementary data 1: Raw data for degradation of individual hydrocarbon components in all treatments. (Separate .xlsx file)

Supplementary information 1: Location and environmental data for stations sampled.

Supplementary information 2: Steps used in MiSeq data analysis.

Supplementary Figure 1: Proportion of hydrocarbons in the model crude oil.

Supplementary Figure 2: Details of natural background bacterial communities in FSC samples.

Supplementary table 1: Treatment conditions in slurry incubation experiments.

Supplementary table 2: Extraction efficiency. % of model oil components recovered after 50 days.

Supplementary table 3: Summary of hydrocarbon groups and the physical characteristics of the components.

Supplementary Table 4: Comparison of degradation rates between sterile incubations and model oil and model oil + dis treatments with ANCOVA and residence times of hydrocarbons based on out data and predicted with linear modeling.

Supplementary information 1. Location and environmental data for stations sampled.

Station	Depth	Location		Temperature	Salinity	DO[1]	Date	Code[2]
	m	Longitude	Latitude	°C	PSU	mmol/l		
FSC500	538	61°07.99’N	02°10.38’W	4.1	35.1	186.3	26/04/2014	NOL_04
FSC1000	994	61°35.00'N	04°15.00'W	-0.7	34.9	194.7	27/04/2014	NOL_07

[1] DO = Dissolved Oxygen.

[2]Code = Marine Scotland long-term stations

Supplementary information 2: Steps used in MiSeq data analysis.

```
Amplicon sequencing of 88 samples produced ~13million reads with read depth
of 30-110K per sample
Analysis done using Biolinux 8 AND Maxwell HPCC

### Pre-processing and data clean up

#join forward and reverse reads to make contigs with pandaseq, max length
of join was 490bp and quality 0.90

$ pandaseq -f seqs/1000-C-0_S5_L001_R1_001.fastq –r seqs/1000-C-
0_S5_L001_R2_001.fastq –w panda/1000-C-0.fasta -L 490 -t 0.90

#this was done for all samples using a loop
#merge the fasta files and make group file for mothur

$ mothur > merge.files(x.fasta, output=merg.fasta)
$ mothur > make.groups(fasta=one.fasta-two.fasta groups=sampleone-
sampletwo)

# edit headers for mothur with

$ sed -i 's/:/_/g' merg.fasta

#remove bad sequences (to short or long, ambiguous bases, and homopolymers
> 8)

$ mothur > screen.seqs(fasta=combined_seqs.fasta, group=mergegroups,
maxambig=0, minlength=465, maxlength=465 maxhomop=8, processors=4)
$ mothur > summary.seqs(fasta=current, processors=4)

#identify unique sequences (saves processor time)

$ mothur > unique.seqs(fasta=current)
$ mothur > count.seqs(name=current, group=current)
$ mothur > summary.seqs(fasta=current, count=current, processors=4)

#Sequence alignment against reference database

$ mothur > align.seqs(fasta=current,reference=silva.align)
$ mothur > summary.seqs(fasta=current, count=current, processors=4)

#clean up alignment and summarise
```

```
$ mothur > screen.seqs(fasta=current, count=current, summary=current,
start=6388, end=25316, processors=4)
$ mothur > summary.seqs(fasta=current, count=current)
$ mothur > filter.seqs(fasta= current, vertical=T, trump=.)

###eliminate any duplicates

$ mothur > unique.seqs(fasta=current, count=current)

#pre cluster sequences allowing 1bp diff per 100 bp in sequence (so diff =
4)

$ mothur > pre.cluster(fasta=current, count=current, diffs=4, processors=1)

###Remove chimeras

$ mothur > chimera.uchime(fasta=current, count=current, dereplicate=t)

#next step remove sequences that were identified as chimeras.

$ mothur > remove.seqs(fasta=current, accnos=current)

###Classify the sequences

$ mothur > classify.seqs(fasta=current, count=current,
reference=greengeens.fasta, taxonomy=greengeens, cutoff=60)

##remove non target DNA (must be error)

$ mothur > remove.lineage(fasta=current, count=current, taxonomy=current,
taxon=Chloroplast-Mitochondria-unknown-Archaea-Eukaryota)

###Pick otus

$ mothur > cluster.split(fasta=current, count=current, taxonomy=current,
splitmethod=classify, taxlevel=4, cutoff=0.15)

$ mothur > make.shared(list=current, count=current)
$ mothur > classify.otu(list=current, count=current, taxonomy=current)
```

Supplementary Figure 1: Proportion of hydrocarbons in the model crude oil.

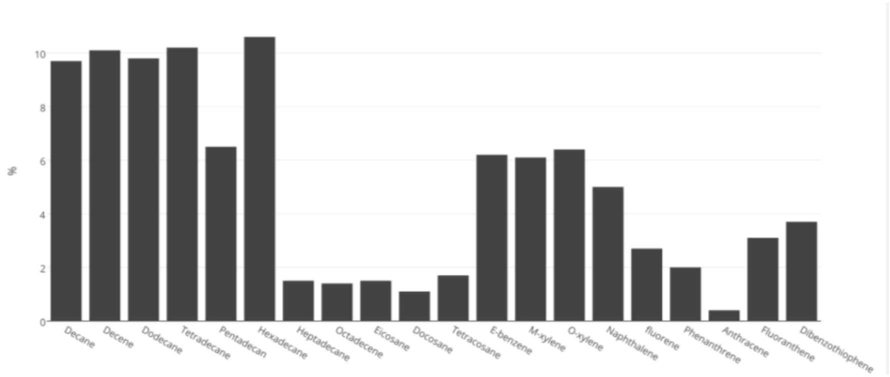

Supplementary Figure 2: Details of natural background bacterial communities in FSC samples.

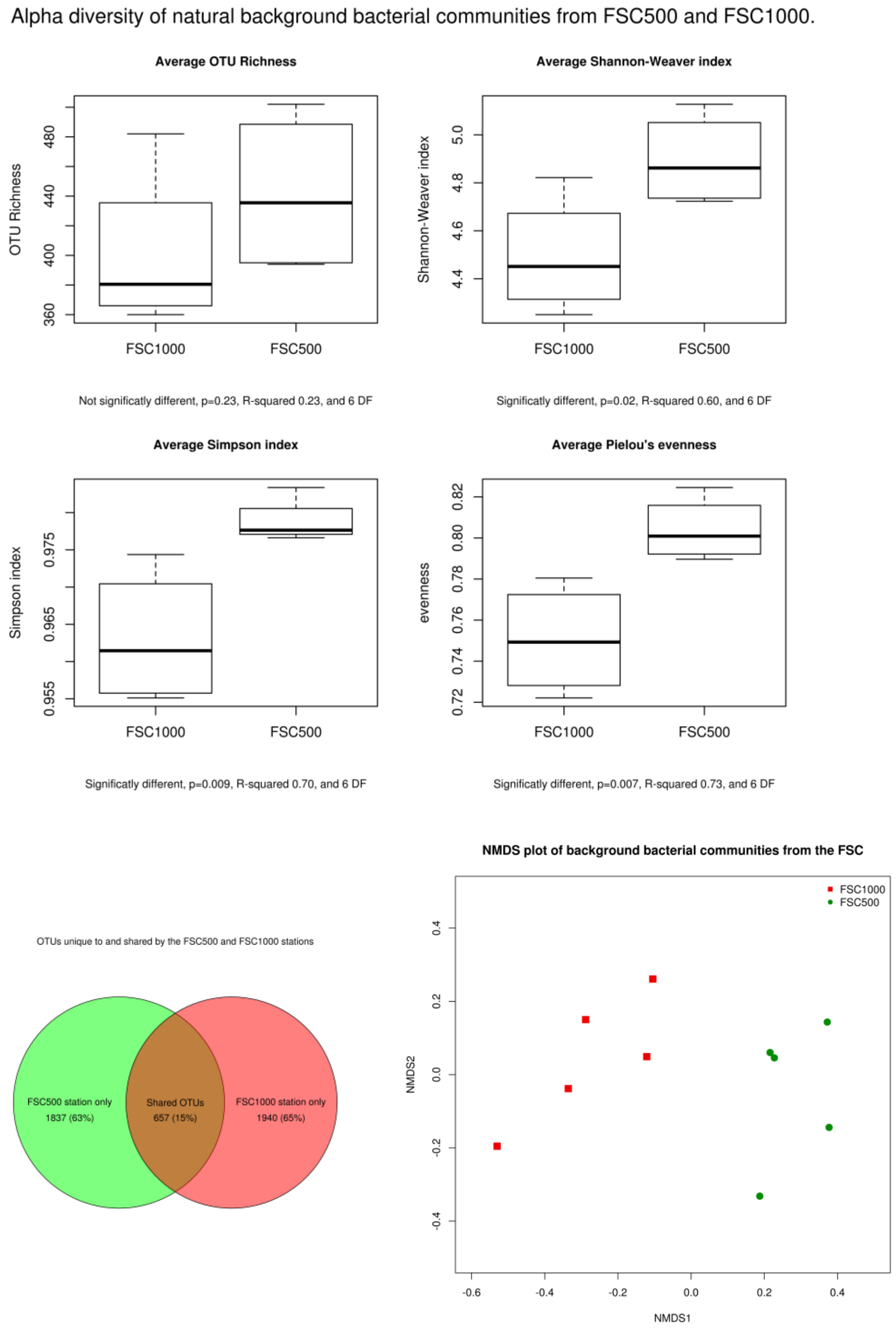

PerMANOVA analysis revealed that the stations were significantly different to each other (p = 0.008, $R^{2} = 0.26$)

Supplementary table 1: Treatment conditions in slurry incubation experiments.

Station	Treatment	Sea Water	Sediment	Oil	Dispersant	Replicates
		ml	g	%	%	
FSC500	Control	15	5	0	0	1
	Steril Control	15	5	2	0	2
	Model oil	15	5	2	0	3
	Model oil + Dis	15	5	2	0.06	3
FSC1000	Control	15	5	0	0	1
	Steril Control	15	5	2	0	2
	Model oil	15	5	2	0	3
	Model oil + Dis	15	5	2	0.06	3

Supplementary table 2: Extraction efficiency. % of model oil components recovered after 50 days.

	FSC500		FSC1000	
component	Oil	Oil + dis	Oil	Oil + dis
n-Decane	93.99 ± 1.07	99.58 ± 5.87	99.15 ± 5.3	98.46 ± 2.59
1-Decene	102.43 ± 3.45	95.55 ± 5.26	95.97 ± 7.4	96.44 ± 7.11
Dodecane	96.32 ± 2.68	98.17 ± 4.12	100.25 ± 5.01	99.59 ± 2.21
Tetradecane	103.23 ± 1.92	98.08 ± 8.1	97.34 ± 8.7	94.31 ± 9.97
Pentadecane	97.78 ± 5.63	102.95 ± 2.99	97.23 ± 3.77	100.52 ± 3.92
Hexadecane	101.9 ± 4.03	103 ± 1.59	95.48 ± 2.92	102.29 ± 2.7
Heptadecane	101.09 ± 6.23	98.66 ± 3.11	92.01 ± 13.76	107.58 ± 4.91
1-Octadencene	93.08 ± 6.11	95.52 ± 5.55	93.46 ± 0.84	101.55 ± 2
Icosane	91.86 ± 3.52	92.27 ± 3.53	86.09 ± 15.25	99.74 ± 11.14
Docosane	101.86 ± 4.79	91.78 ± 6.45	91.93 ± 14.91	94.81 ± 13.68
Tetracosane	103.28 ± 6.06	77.55 ± 10.72	87.82 ± 9.39	100.58 ± 6.16
E-benzene	**65.06 ± 28.27**	**62.63 ± 28.07**	**64.51 ± 22.64**	**70.42 ± 29.14**
M-xylene	**58.92 ± 18.49**	**71.87 ± 32.48**	**71.6 ± 24.07**	**62.31 ± 23.74**
O-xylene	**57.08 ± 19.1**	**71.49 ± 35.28**	**60.29 ± 25.78**	**71.37 ± 28.1**
Napthalene	85.66 ± 4.4	95.41 ± 3.96	108.68 ± 7.02	96.5 ± 7.35
Fluorene	97.02 ± 8.52	98.59 ± 5.56	93.12 ± 4.12	100.86 ± 6.17
Phenathrene	92.64 ± 7.4	100.65 ± 2.27	93.04 ± 7.25	99.33 ± 4.49
Anthracane	95.72 ± 4.31	101.38 ± 4.5	95.48 ± 5.93	99.97 ± 6.21
Fluoranthene	93.29 ± 7.59	85.33 ± 10.72	101.89 ± 9.27	96.43 ± 6.26
Dibenzothiophene	87.81 ± 2.91	94.02 ± 5.14	93.42 ± 9.01	102.91 ± 11.23
**total**	**93.12 ± 5.1**	**90.86 ± 1.08**	**95.44 ± 5.87**	**93.50 ± 5.36**

Supplementary Table 3. Summary of the aliphatic hydrocarbon groups predicted by PCA and the physical characteristics of the components.

Group	Component	Carbons	log K_{ow}	Solubility (mg l-1)	Class
Aliphatic					
1	n-Decane	10	5.01	0.052	Straight
	1-Decene	10	5.7	0.57	Straight
2	Pentadecane	15	7.71	0.000076	Straight
	Hexadecane	16	8.2	0.0009	Straight
	Heptadecane	17	8.69	0.000294	Straight
	Icosane	20	10.16	0.0019	Straight
	Docosane	22	11.15	9.37E-07	Straight
3	Dodecane	12	6.1	0.0037	Straight
	Octadecene	18	9.04	0.000126	Straight
4	Tetracosane	24	12.13	9.25E-08	Straight

Supplementary Table 4: Comparison of degradation rates between sterile incubations and model oil and model oil + dis treatments with ANCOVA and residence times of hydrocarbons based on out data and predicted with linear modeling. †Stars show significant difference level: '***' $p < 0.001$ '**' $p < 0.01$ '*' $p < 0.05$.

Station	Group	Rate comparisons with ANCOVA				Predicted residence time		
		oil v sterile		oil + dis v sterile		Extrapolated Residence time		
		R^2	significance†	R^2	significance†	Days	R^2	significance†
Aliphatic								
FSC500	1	0.91	***	0.81	***	68.56	0.87	***
	2	0.44	**	0.74	**	99.39	0.89	**
	3	0.87	**	0.92	***	58.54	0.82	***
	4	0.43	**	0.83	***	64.35	0.89	**
FSC1000	1	0.68	**	0.80	**	105.03	0.79	**
	2	0.46	*	0.82	***	157.51	0.29	*
	3	0.45	*	0.72	**	148.62	0.39	**
	4			0.34	*	Not predictable		
Aromatic								
FSC500	Naphthalene	0.89	***	0.94	***	57.76	0.92	***
	Fluorene	0.70	*	0.78	**	57.02	0.79	***
	Phenanthrene	0.77	**	0.81	***	58.14	0.60	***
	Anthracene	0.64	**	0.69	*	57.04	0.75	***
	Fluoranthene	0.50	*	0.74	**	84.99	0.89	**
	Dibenzothiophene	0.43	*	0.56	**	58.84	0.50	***
FSC1000	Naphthalene	0.34	**	0.63	**	153.82	0.39	*
	Fluorene	0.16	**	0.68	**	NA	NA	
	Phenanthrene	0.48	**	0.75	**	86.04	0.47	**
	Anthracene	0.39	NA	0.63	*	NA	NA	
	Fluoranthene	0.07	NA	0.50	*	NA	NA	
	Dibenzothiophene	0.16	NA	0.70	**	NA	NA	
